# Social Learning and Preferences in Adolescents With Conduct Problems and Varying Levels of Callous-Unemotional Traits

**DOI:** 10.1016/j.jaacop.2023.12.008

**Published:** 2024-01-12

**Authors:** Anne Gaule, Leonardo Bevilacqua, Lucas Molleman, Wouter van den Bos, Anna C. van Duijvenvoorde, Ruth Roberts, Christopher R. Pease, Eamon McCrory, Essi Viding

**Affiliations:** a University College London, United Kingdom; b University of Amsterdam, the Netherlands; c Max Planck Institute for Human Development Berlin, Germany; d Amsterdam Brain and Cognition, University of Amsterdam, the Netherlands; e Leiden University, the Netherlands, and Leiden Institute for Brain and Cognition, the Netherlands

**Keywords:** aggression, conduct problems, callous-unemotional traits, social learning, collaboration

## Abstract

**Objective:**

Learning to successfully navigate the social world, in particular when to trust others and how to work together with them, is crucial to well-adjusted social development. This is especially the case during adolescence, when individuals are undergoing significant biological and social changes. Adolescents with conduct problems (CP) tend to have difficulties in social relationships and to display aggressive behaviors as well as reduced cooperation with others. This pattern appears to be particularly pronounced in adolescents with CP and high callous-unemotional traits (CP/HCU). However, very little is currently known about the mechanisms that might drive reduced cooperative behaviors in adolescent CP, and whether these differ for individuals with high vs low levels of CU traits.

**Method:**

We used a series of economic games to assess how adolescents with CP/HCU (n = 46), CP, and lower levels of CU traits (CP/LCU) (n = 46), and typically developing adolescents (TD) (n = 59) interacted with social (human) and non-social (computer) partners that varied in their degree of cooperation (trustworthy vs untrustworthy and friendly vs unfriendly), and whether this related to group differences in social preferences (aversion to inequality) and prior beliefs.

**Results:**

Adolescents with CP (both HCU and LCU) had more difficulty than TD adolescents in differentiating between trustworthy and untrustworthy social environments in our task. Adolescents with CP/LCU also had more difficulty coordinating with friendly and unfriendly social partners to produce rewarding outcomes than TD adolescents. Surprisingly, we saw no relationship between participants’ inequality aversion or prior beliefs and social learning in our games.

**Conclusion:**

These findings indicate that, under controlled experimental conditions, adolescents with CP have more difficulty learning to differentiate between social environments that vary in cooperation, particularly adolescents with CP/LCU. These findings were not explained by inequality aversion or prior beliefs. Our findings also raise important questions regarding methods used to understand the mechanisms underlying social behaviors in adolescents with CP.

## Plain language summary

In this interesting study of 151 boys between the ages of 11 and 16 years, authors used economic games to investigate how adolescents with conduct problems learn to cooperate with social (human) and nonsocial (computer) partners under various social environments. The authors found that, relative to peers without any conduct problems, adolescents with conduct problems had difficulty gauging trustworthiness in social environments and coordinating with social partners to achieve rewarding outcomes. These findings were not explained by social preferences or prior beliefs. Adolescents with conduct problems and conduct problems with low callous unemotional traits may have more difficulty determining who is trustworthy and friendly, respectively, which may lead to reliance on aggression to cope with these difficulties.

Humans inhabit an environment that heavily relies on social interaction. The ability and willingness to trust and collaborate with others have been identified as crucial factors in effectively navigating this environment and achieving positive social adjustment.^1^^,^^2^ Well-adapted social behavior has, in turn, been associated with various favorable developmental outcomes such as improved physical health, educational achievements, and long-term mental well-being.^3^^,^^4^ Children and adolescents with conduct problems (CP) present with aggressive behaviors, have trouble adjusting to social norms, and have difficulties in maintaining healthy relationships with family members and peers.^5^, ^6^, ^7^ CP represents one of the most common reasons for referral for child and adolescent mental health services in the United Kingdom and is associated with a range of adverse individual and societal outcomes including poor mental and physical health, higher likelihood of leaving school without qualifications, and involvement in the criminal justice system.^8^^,^^9^

It has recently been argued that impairment in a learning system that has evolved to promote and protect collaborative behaviors is an important factor for the development of aggressive behaviors such as those exhibited in CP.^10^ In line with this, research indicates that aggression and CP in adolescence are characterized by varied patterns of maladaptive social interactions including increased engagement in bullying behaviors, reduced engagement in prosocial behaviors, and difficulties with social problem solving and conflict management.^5^^,^^11^, ^12^, ^13^ The maladaptive social interactions that characterize the relationships of children and adolescents with CP have far-reaching consequences for both individual and collective welfare, yet we know remarkably little about how cognitive processes supporting social interactions may differ between those with or without CP. Because interventions for CP typically focus on improving social relationships,^14^ it is critical to increase our understanding of cognitive processes that might constrain how such interventions are developed, framed, and applied.

Furthermore, research indicates that social difficulties in CP may stem from partially divergent social–cognitive profiles depending on whether an individual with CP also displays high or low levels of callous-unemotional traits (CP/HCU vs CP/LCU). If particular social information processing biases characterize only a subgroup of children with CP, then any intervention development would have to take this into account. Adolescents with CP/HCU (a particularly vulnerable group at risk for developing psychopathy in adulthood^15^, ^16^, ^17^) have difficulty empathizing with others and also show reduced responsiveness to social cues that promote affiliation.^18^, ^19^, ^20^ There is some evidence that they demonstrate especially reduced prosocial behavior (relative to adolescents with CP/LCU), and also endorse non-cooperative goals such as dominance, demanding respect from others, and revenge in situations of social conflict.^21^^,^^22^ Adolescents with CP/LCU do not appear to have the same difficulties empathizing and affiliating with others. However, these adolescents appear to have more trouble than those with CP/HCU with flexible social problem solving, and may have a tendency to stick with initial judgments.^23^^,^^24^ Thus, although poor social adjustment and reduced collaborative behavior appear to be characteristic of CP generally, the reasons underlying this might vary for different subgroups of adolescents with CP. We also know very little about the mechanisms that might underlie poor social adjustment in CP.

Westhoff *et al.*^25^ suggest that decisions to cooperate in typically developing (TD) adolescents are underpinned by at least 3 factors, all of which might contribute to individual differences in engagement in cooperative behaviors: (1) social preferences, (2) prior expectations, and (3) updating of expectations based on feedback. Social preferences refer to the degree to which individuals care about relative outcomes, for example, getting more or less than other people.^26^ Advantageous inequality aversion refers to the dislike of getting more than others, and disadvantageous inequality aversion refers to the dislike of getting less than others. Prior expectations refer to people’s perceptions of what most people do.^27^ These expectations help inform our social decisions by allowing us to predict how we think someone else will respond to our choices or behaviors: for example, whether someone else will reciprocate if we ourselves cooperate. Updating of expectations refers to being able to change our already held expectations in light of new information. For example, if a peer continuously violates our trust, it is likely that we will re-evaluate their trustworthiness based on this behavior. Westhoff *et al.* observed that differences in both social preferences and updating of expectations provided a mechanistic explanation for age-related differences in social decision making.^25^ More specifically, adolescents in Westhoff *et al.*’s study showed lower levels of cooperation than adults, and this was partly explained by higher disadvantageous inequality aversion. Furthermore, adolescents in their sample were able to update their expectations more readily than adults, and were more likely to change behavioral strategies based the outcomes of their decisions.

Research to date has provided initial evidence that adolescents with CP demonstrate atypicalities in at least some of these factors identified as important for cooperative decision making. For example, adolescents with CP appear to be highly sensitive to unfair offers in economic games relative to TD peers (ie, they demonstrate increased disadvantageous inequality aversion).^28^ CP/HCU in particular appears to be associated with an increased willingness to receive more than one’s own fair share in such games (ie reduced advantageous inequality aversion).^22^^,^^29^^,^^30^ In terms of prior beliefs, it has been argued that CP is characterized by reduced epistemic trust, that is, a reduced ability to distinguish (or come to know) trustworthy from untrustworthy communicators.^10^^,^^31^ Combined with evidence of a hypervigilance to threat in adolescents with CP,^32^ it is possible that these adolescents have prior beliefs that others will not cooperate with them (although, to our knowledge, this is yet to be formally investigated). Finally, adolescents with CP demonstrate difficulty with (non-social) reinforcement-based learning.^15^^,^^33^ If these difficulties extend to the social domain, the ability of individuals with CP to update expectations in response to new information may be compromised. This is supported by evidence that adolescents with CP use fewer cooperative strategies following a partner’s defection in economic games,^34^ and those with CP/LCU demonstrate a tendency to stick with initial judgments rather than updating responses to new social information.^23^

Overall, research indicates that adolescents with CP demonstrate reduced cooperative behavior, and may also show atypicalities in cognitive processes that are thought to underlie cooperative behavior. However, to our knowledge, no study has directly addressed how social learning in adolescents with CP relates to cooperative behaviors in different social environments, and whether this varies with high vs low CU traits. Furthermore, no study has used measures that would enable direct testing of how group differences in social preferences (aversion to inequality), prior beliefs regarding others’ cooperative behavior, and updating of these beliefs relate to social learning in adolescents with CP/HCU and CP/LCU compared to TD peers. In the current study, we explore these questions using several well-established, incentivised economic games (the Trust Game, Coordination Game, and child-friendly versions of the Dictator and Ultimatum Games).

Based on research suggesting reduced cooperation and epistemic trust in adolescent CP, we hypothesized that participants with CP/HCU and CP/LCU would have more difficulty in learning to adjust to cooperative and uncooperative social environments than TD participants (both social environments that vary in trust, and environments that vary in degree of social coordination required). We also hypothesized that these differences would be explained by a combination of group differences in (1) social preferences (2) prior expectations, and (3) belief updating. We further hypothesized that the factors driving behavioral differences between CP and TD adolescents may vary based on whether the adolescents with CP also have high CU traits. Specifically, we hypothesised that adolescents with CP, particularly CP/HCU, would be less averse to advantageous inequality (based on research indicating that these groups are less averse to having more than one’s fair share), and that all adolescents with CP would demonstrate reduced disadvantageous inequality aversion (based on research indicating that CP is associated with high sensitivity to unfair offers in economic games). In line with the evidence that CP is associated with reduced epistemic trust and hypervigilance to threat, we hypothesised that adolescents in CP groups would have stronger prior expectations that others would be uncooperative (both in terms of trust, and social coordination). Finally, based on research indicating disrupted reinforcement learning in adolescents with CP, as well as research indicating a tendency to stick to simple strategies in social games, we hypothesized that adolescents with CP, perhaps especially those with CP/LCU, might show disrupted expectation updating.

## Method

### Sample and Procedure

This study involved 155 boys between the ages of 11 and 16 years from mainstream schools and specialized alternative provision schools for adolescents with behavioral difficulties in London and the Home Counties. To identify pupils who were eligible to take part, teachers were given screening questionnaires to do the following: (1) classify current CP, (2) conduct a dimensional assessment of CU traits, (3) screen for commonly co-occurring symptoms with CP, and (4) provide information regarding specialist education provision. Exclusion criteria included a parent or teacher report of a formal autism spectrum disorder diagnosis or of the presence of severe learning difficulties. Four CP participants were removed from our sample on the basis of these criteria. Our final sample for descriptive and main experimental analyses therefore included 151 boys (46 CP/HCU, 46 CP/LCU, 59 TD). For details on group assignment, as well as consent/assent procedures, please see Supplement 1, available online. Sample characteristics are reported in Table 1. The current study was approved by the University College London Research Ethics Committee (Project ID number: 0622/001).

**Table 1:** Group Matching and Selected Participant Characteristics Data

	CP/HCU (n = 46)			CP/LCU (n = 46)			TD (n = 59)			*p*	Post hoc^a^
	Mean (SD)	Min-max	N_complete_	Mean	Min-max	N_complete_	Mean	Min-max	N_complete_		
Wechsler Abbreviated Scale of Intelligence (Full Score)^b^	86.13 (8.97)	72.00–111.00	45	88.46 (11.90)	67.00–120.00	46	90.34 (10.97)	70.00–114.00	58	.023^c^	2<3
Age, y^d^	14.41 (1.23)	11.70–16.49	46	13.67 (1.43)	11.55–16.30	46	14.08 (1.19)	11.77–16.85	59	.145	—
Pubertal Stage^b^^,^^e^^,^^f^	0:4:20:19:2	4.33–12.00	45	1:13:18:14:0	3.00–11.00	46	1:6:26:23:2	3.00–12.00	58	.181	—
CASI Conduct Disorder^d^^,^^g^	9.79 (4.98)	3.00–25.00	46	6.74 (3.27)	3.00–18.00	45	0.31 (0.68)	0.00–2.00	59	<.01^c^	1<2<3
ICU^d^^,^^g^	47.15 (6.69)	38.61–63.00	46	30.28 (6.14)	14.00–38.40	46	19.47 (6.95)	2.00–37.00	59	<.001^c^	1<2<3
Alcohol Use and Disorders^b^^,^^e^^,^^h^	40:5:1	0.00–20.00	46	45:0:1	0.00–16.00	45	57:2:0	0.00–9.00	59	.098	—
Drug Use and Disorders^b^^,^^e^^,^^i^	37:9	0.00–21.00	46	41:5	0.00–20.00	45	56:3	0.00–25.00	58	.066	—
SDQ Hyperactivity^d^	7.78 (2.38)	2.00–10.00	46	6.40 (2.73)	1.00–10.00	45	2.48 (2.26)	0.00–9.00	58	<.001^c^	1<2<3
SDQ Emotional Problems^d^^,^^g^	3.37 (2.82)	0.00–10.00	46	3.51 (2.67)	0.00–10.00	45	1.28 (1.88)	0.00–9.00	58	<.001^c^	1<2, 1<3
SDQ Peer Problems^d^^,^^g^	3.61 (2.46)	0.00–9.00	46	2.82 (1.81)	0.00–8.00	45	1.35 (1.58)	0.00–5.00	58	<.001^c^	1<2, 1<3

Note: Where not stated, analyses were performed using 1-way analysis of variance and post hoc tests, and were Bonferroni corrected for multiple comparisons. Full analysis of participant characteristics data reported in Table S2, available online. CASI *=* Child and Adolescent Symptom Inventory (see Supplement 1, available online); CP/HCU = conduct problems and high levels of callous-unemotional traits; CP/LCU = conduct problems and low levels of callous-unemotional traits; ICU = Inventory of Callous and Unemotional traits (see Supplement 1, available online); n = number of participants with complete measure; SDQ = Strengths and Difficulties Questionnaire (see Supplement 1, available online); TD = typically developing.

a 1 = TD, 2 = CP/LCU, 3 = CP/HCU.

b Measure obtained at testing phase, child report.

c Results for comparisons less than or equal to this threshold.

d Measure obtained at screening phase, teacher report.

e Assessed via χ^2^ test, *p* value computed for a Monte Carlo test.^35^ Post hoc tests were Bonferroni corrected for multiple comparisons.

f Counts for Pubertal Stages (Pre-pubertal: Early-pubertal: Mid-pubertal: Advanced-pubertal: Post-pubertal)

g Assessed via 3 pairwise Mann–Whitney *U* tests because of violation of analysis of variance assumptions. Directionality inferred through visual inspection of means.

h Counts for Alcohol Use and Disorders risk categories (Low Risk: Increasing Risk: Higher Risk: Possible Dependence). For more information on our measures of substance use, see Supplement 1 and Supplement 2, available online).

i Counts for Drug Use and Disorders risk categories (Low Risk: Possible Drug Problems). For more information on our measures of substance use, see Supplement 1 and Supplement 2, available online).

Groups were similar with respect to IQ, pubertal status, substance use (Table 1; full reporting in Supplement 2, measures described in Supplement 1, available online). Differences were observed between groups in average age as well as variables that commonly occur with CP (substance use, hyperactivity, peer problems, emotional problems^13^, ^36^, ^37^; analyses reported in Supplement 2, available online). We were unfortunately unable to compare whether groups were similar with respect to ethnicity due to low return rates of parent questionnaires (where ethnicity was assessed; more information in Supplement 2, available online).

### Economic Games

**Figure 1:**
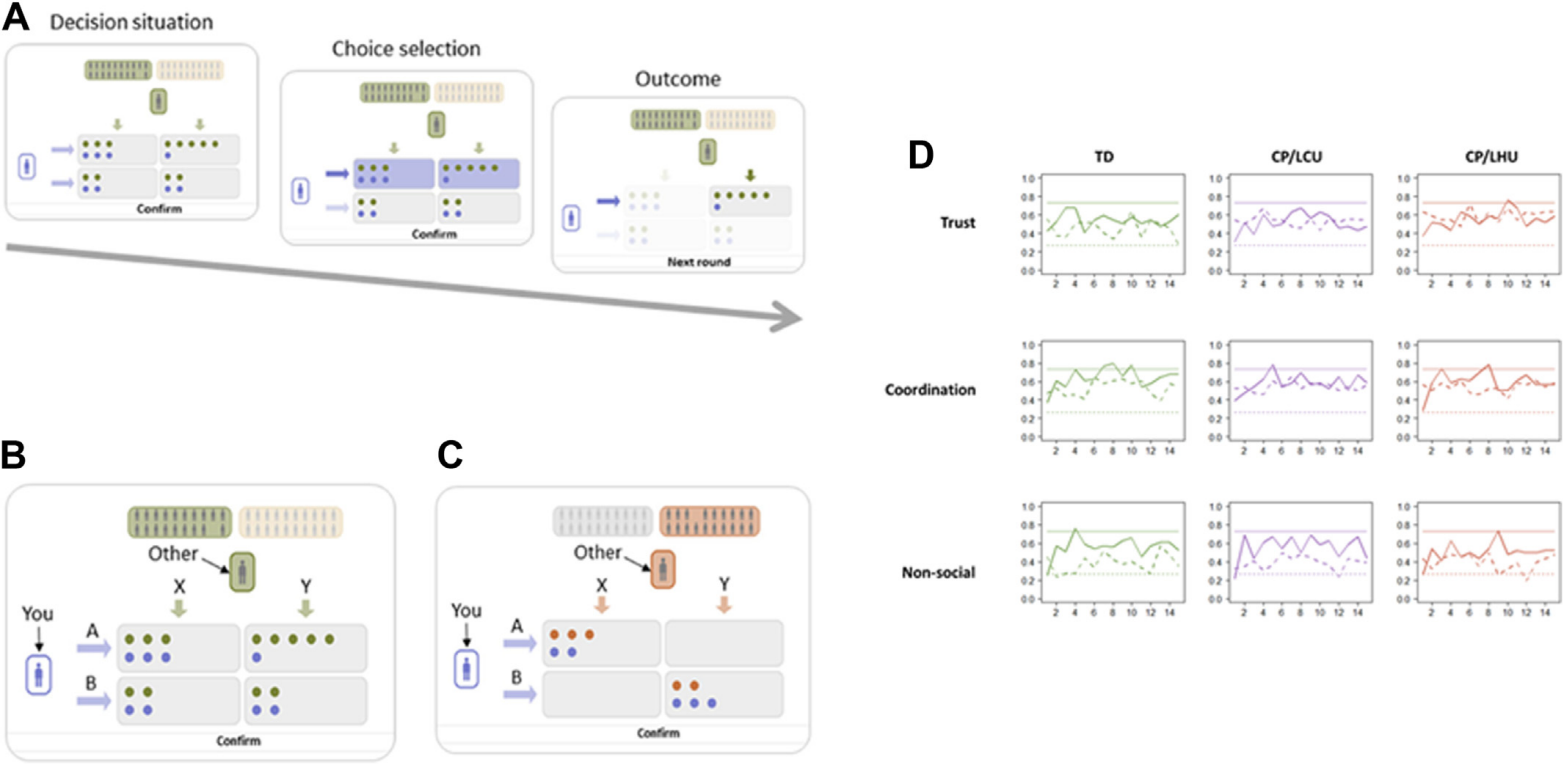
Illustration of Tasks and Participant Choice Behavior ***Note:****(A-C) Reproduced from Westhoff* et al., *2020**^25^**(published under Creative Commons license CC BY:*http://creativecommons.org/licenses/by/4.0/*) (A) Task assessing individuals’ ability to adapt to social environments characterized by cooperation and lack of cooperation. In an example trial, a participant (depicted as a purple stick figure on the left) is presented with 2 rows of boxes labeled A and B and can choose between them. After making their choice, the participant is shown the predetermined choice (X or Y) of another player (depicted as a gray stick figure at the top). The background color of the other player indicates the social environment to which they belong. The combined choices of the participant and the other player determine the monetary outcome for both individuals, represented by the number of dots in their respective colors. (B) In the Trust Game, participants interact with players from either a “Trustworthy” environment (who tend to choose X) or an “Untrustworthy” environment (who tend to choose Y). To maximize their own monetary payoffs, participants are encouraged to trust (choose A) a player from the Trustworthy environment and to withhold trust (choose B) from a player from the Untrustworthy environment. In this setup, only disadvantageous inequality aversion may play a role in decision making. (C) In the Coordination Game, participants aim to maximize their monetary payoffs by aligning their choices with those of their fellow players. Similar to the Trust Game, the social environments in this game also vary in terms of cooperation levels, which can be observed through the players’ tendencies to choose either X or Y. Positive outcomes are achieved by coordinating on either of the outcomes (A, X) or (B, Y). Specifically, participants maximize their own monetary payoffs by accepting a disadvantage (choosing A) when faced with a player from the “Unfriendly” environment and accepting an advantage (choosing B) when faced with a player from the “Friendly” environment. In this game, both advantageous and disadvantageous inequality aversion may influence social decision making.****(D)****Participant choices over trials per social environment and group (conduct problems [CP] and high callous-unemotional traits [CP/HCU]), CP and low CU traits (CP/LCU), and typically developing (TD). Solid and dotted lines show fractions of choices for A when matched with a partner from cooperative and uncooperative social groups, respectively. Horizontal lines show the proportions of X and Y chosen by partners from cooperative and uncooperative social groups, respectively. In the Trust game, both CP/HCU and CP/LCU groups were poorer than the TD group at differentiating trustworthy (cooperative) and untrustworthy (uncooperative) environments. In the Coordination game, only the CP/LCU group significantly differed from the TD group in their ability to differentiate friendly (cooperative) and unfriendly (uncooperative) environments. Groups did not differ in their ability to differentiate cooperative and non-cooperative social partners in a non-social environment (more information on our non-social task in*Supplement 1*, available online).*

The current study used the same protocol as Westhoff *et al.*^25^ The study involved participants playing a series of well-established, incentivized, economic games. The Trust Game and the Coordination Game were used to evaluate how participants adjusted to different social environments (Figure 1A-C). Prior to these social economic games, participants also completed a non-social learning task. Although our main research questions are centered on social decision making, this task was included to examine behavioral adjustment in a simple learning context. In the non-social task, participants interacted with computers as their partners, and only the participants were eligible to receive payoffs. Full detail on all tasks, as well as on study procedure, can be found in Supplement 3, available online.

### Social Preferences

Social preferences (ie, advantageous and disadvantageous inequality aversion) were measured in modified versions of 2 separate tasks: a Dictator Game, and an Ultimatum Game. Both games were adapted to be short and child friendly.^25^^,^^38^ More detail is provided in Supplement 3, available online.

### Prior Expectations

Participants’ prior expectations of others’ behavior were assessed before starting the Trust Game and before the Coordination Game. Participants were asked “Suppose that there are 10 other players. How many out of these 10 do you believe will choose Option X?”, which was designed to elicit their preconceived expectations regarding the trustworthiness of others (in the Trust Game), or their expectations regarding others’ inclination to seek an advantage over someone else (in the Coordination Game). The participants' responses ranged from 0 to 10.

### Belief Updating

We used computational reinforcement learning models to model the process of updating expectations between interactions in the Trust and Coordination games. These models used a learning rate parameter, which determined the extent to which an expectation violation influenced subsequent expectations and, consequently, decision making.^39^

## Results

We first looked at choice behavior over the course of the Trust and Coordination games to assess whether adolescents with CP/HCU, CP/LCU, and TD adolescents in our sample adjusted choice behavior to different social environments. We also examined choice behavior in a simple non-learning context. Choice behavior in each game was examined by fitting binomial generalized linear mixed models (GLMM) to the participants’ binary choices (A or B) (Figure 1; Table S1 and Table S2, available online).

**Figure 2:**
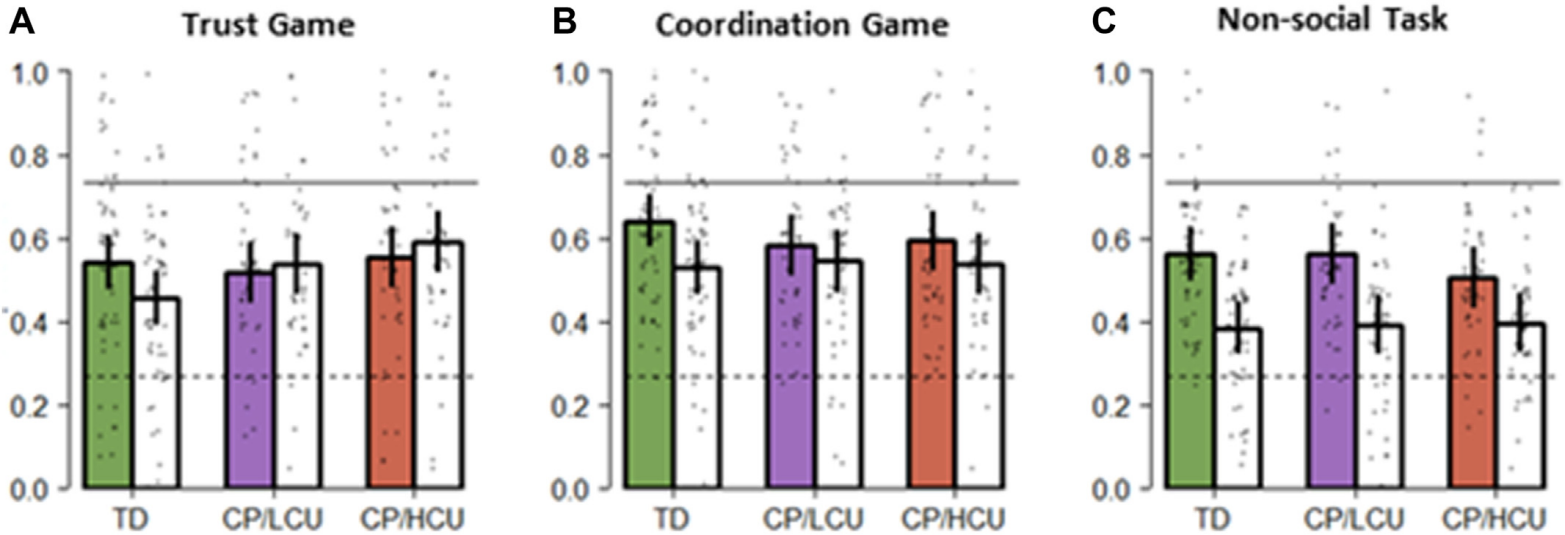
Participant Choices by Decision Setting Participant Group and Interaction Partner Group ***Note:****Proportions choices for A for each of the decision settings (panels), participant groups, and groups of interaction partners (colored bars: partner tended to play X; white bars: partner tended to play Y). Bars show means ± SEs of proportions for A for each individual. Individuals are also indicated with gray dots. CP/HCU = conduct problems and high levels of callous-unemotional traits; CP/LCU = conduct problems and low levels of callous-unemotional traits; TD = typically developing.*

Analysis of choices in the Trust Game revealed that both CP/HCU and CP/LCU participants had a significantly lower ability than TD participants to distinguish between trustworthy and untrustworthy social environments (participant group by partner group interaction; CP/HCU vs TD, *B* = –0.565, *p* < .001; CP/LCU vs TD, *B* = –0.478, *p* = .002), but that CP groups did not significantly differ from one another (CP/HCU vs CP/LCU, *p* = .593; differences between colored and white bars, Figure 2A; Table S1, available online).

In the Coordination game, adolescents with CP/LCU were less able (to a statistically significant extent) to distinguish between friendly and unfriendly social environments (CP/LCU vs TD, *B* = –0.322, *p* = .031) (Fig 2B). Although adolescents with CP/HCU were also poorer at this than TD adolescents, they did not differ statistically from either group (CP/HCU vs TD, *B* = –0.247, *p* = .101; CP/HCU vs CP/LCU, *B* = 0.075, *p* = .635) (Table S1, available online).

Groups did not significantly differ in the ability to distinguish between environments in a non-social context, although we saw a trend toward our CP/HCU group being poorer in this distinction than our TD group (CP/HCU vs TD, *B* = –0.283, *p* = .053; CP/LCU vs TD, *B* = –0.037, *p* = .798; CP/HCU vs CP/LCU, *B* = –0.246, *p* = .112) (Table S2, available online). Full details on model specification, are available in Supplement 4, available online.

### Social Preferences, Prior Beliefs, and Belief Updating

**Figure 3:**
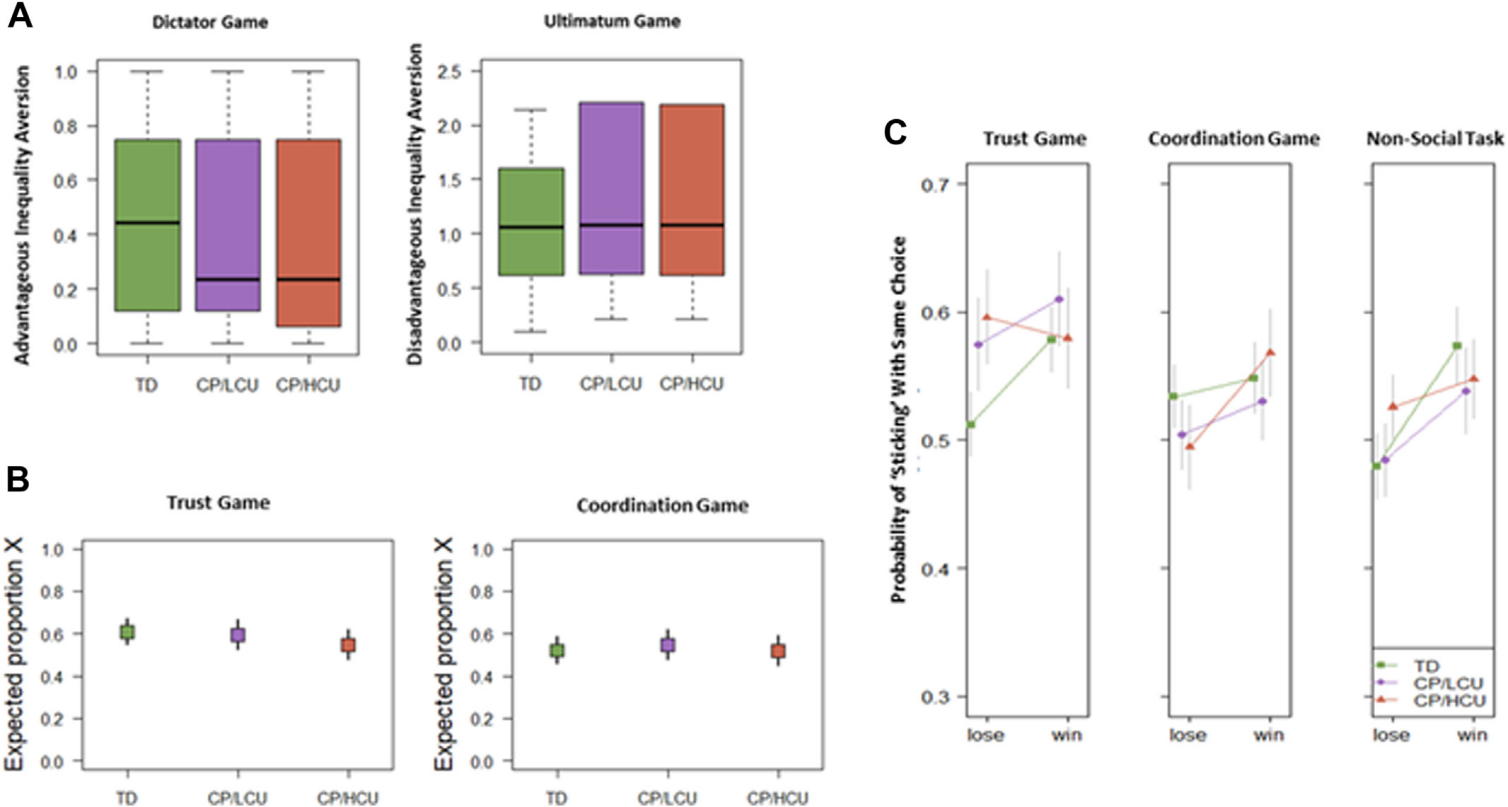
Social Preferences, Prior Beliefs, and Responses to Different Outcomes Across Groups ***Note:****(A) Social preferences are represented by indifference points in in the Dictator Game, which measure advantageous inequality aversion, and the Ultimatum Game, which measures disadvantageous inequality aversion (details of calculation of these measures can be found in*Supplement 3*, “Measuring Social Preferences,” available online). (B) Prior expectations are assessed for the Trust Game, where higher values of “Expected proportion X” indicate greater expectations of trustworthiness in others, and for the Coordination Game, where higher values of “Expected proportion X” indicate greater expectations of friendliness and consideration for others’ payoffs rather than prioritizing their own. (C) Responses to social interaction outcomes. Panels show, for each of the interaction contexts, the average probabilities (±1 SEM) of participants staying with the same option (A or B) after experiencing a “loss” (defined as choosing the option that did not lead to the best outcome, given the choice of the partner; ie, choosing A when the partner chose Y, or choosing B when the partner chose X), or a “win” (defined as choosing the option that led to the best outcome; ie, choosing A when the partner chose X, or choosing B when the partner chose Y). CP/HCU = conduct problems and high levels of callous-unemotional traits; CP/LCU = conduct problems and low levels of callous-unemotional traits; TD = typically developing.*

We next looked at factors that may contribute to group differences in adapting to different social environments, starting with social preferences (Fig. 3A).

We observed that CP/LCU and particularly CP/HCU seem to be less averse to advantageous inequality (mean disutility for each unit being ahead: TD: 0.47; CP/LCU: 0.40; CP/HCU: 0.29). However, groups did not differ significantly from one another (linear model fitted to participants’ advantageous inequality aversion (TD vs CP/HCU: *p* = .268; TD vs CP/LCU: *p* = .649; CP/HCU vs CP/LCU, *p* = 0.379)) (Figure 3A; Table S3, available online).

All groups demonstrated strong disadvantageous inequality aversion (mean disutility for each unit being behind: TD: 0.88; CP/LCU: 0.85; CP/HCU: 0.87), which was significantly stronger than advantageous inequality aversion (paired *t* test: *p* < .001). However, groups did not differ significantly from one another in this respect (linear model fitted to participants’ advantageous inequality aversion: TD vs CP/HCU: *p* = 0.687; TD vs CP/LCU: *p* = .608; CP/HCU vs CP/LCU, *p* = .920)) (Figure 3A; Table S4, available online).

We next explored group differences in prior beliefs (Fig. 3B). A logistic regression revealed no group differences in prior beliefs regarding others’ trustworthiness (all *p* > .755) (Table S5, available online) or their preference to coordinate on the action that benefited the participant most (ie, to choose option X) (all *p* > 0.104). Model specification for analysis of both prior beliefs and social preferences can be found in Supplement 4, available online.

Finally, we used computational models (extended reinforcement learning models; more detail in Supplement 4, available online) to examine how participants updated their expectations in different social environments. However, estimated sensitivities in these models were so low that learning rates could not be interpreted for any of our participant groups. We therefore resorted to a simpler analysis based on heuristics of win-stay lose-shift, as discussed below.

### Exploratory Analyses

To explore whether groups differed in the heuristics that they used in our Trust and Coordination games, as well as in the non-social task, we examined the extent to which they used a “win–stay, lose–shift” strategy. To this end, we fitted a logistic GLMM to predict participants’ decisions to stick with the same choice, based on the outcome of the previous interaction (for each environment) (Figure 3C, Table S6, available online).

We observed that, for both good and bad outcomes in the previous interaction, probabilities of sticking with the same option were close to 50%. This means that, overall, behavior was quite erratic (note that a perfect win–stay, lose–shift strategy would have a y-value of 0 for “lose” and 1 for “win”). That said, all groups were more likely to stick with the same choice following a win in the non-social task and the Trust Game (*B* = 0.454, *p* < .001; *B* = 0.312, *p* = .004 respectively) (Table S6, available online). This was not seen in the Coordination game (*p* > .420). In the Trust Game, the HCU group had an anti-correlated pattern that differed significantly from that of the TD group (ie, they were more likely to stick with the same group following a loss, and shift to a new group following a win; *B* = –0.420, *p* = .012). In the Coordination Game, we observed no significant group differences, although the CP/HCU participants were the most sensitive to the outcome of the previous round. The CP/LCU group did not differ from other groups in their heuristics (all *p* > .10).

### Covariate Analyses

As groups differed in age, we reran our key models exploring adaptation to different social environments with age as a covariate (Tables S7 and S8, available online). This did not change any of our findings, indicating that variance in age did not account for groups’ abilities to differentiate between (un)trustworthy social environments and coordinate with (un)friendly social environments. We also ran 4 additional models predicting adaptation to each environment with Strengths and Difficulties Questionnaire teacher ratings of hyperactivity, emotional difficulties, and peer problems (ie variables that differed between groups) included as covariates (Tables S7, S8, available online). None of these variables contributed to variance in groups’ abilities to differentiate (un)trustworthy social environments. However, including these variables as covariates removed the difference between CP/LCU and TD participants in the ability to coordinate with (un)friendly social partners, which indicates that this difference may relate to other factors that are important for the clinical presentation of CP.

## Discussion

In the current study, we examined whether adolescents with conduct problems and high vs low levels of callous-unemotional traits (CP/HCU vs CP/LCU) differ from typically developing (TD) adolescents in their ability to learn to adjust to social environments that vary in their degree of cooperation. We further assessed whether group differences in social preferences (inequality aversion), prior expectations regarding others’ behavior, and updating of expectations might provide a mechanistic explanation of observed differences in behavioral adjustments beween groups. Participants played economic games capturing trust and social coordination, 2 important cooperative behaviors. In line with our hypotheses, we observed differences between the CP and TD groups in their ability to learn to adjust to different social environments, resulting in reduced cooperation in these groups. Interestingly the extent of these differences varied based on the social environment in question. Although both CP/HCU and CP/LCU participants were considerably poorer at differentiating trustworthy and untrustworthy environments than were TD participants, only CP/LCU participants differed statistically from TD participants in their ability to differentiate friendly and unfriendly social environments. Surprisingly, however, we observed no group differences in any of the purported factors underlying cooperative behaviors that we investigated. These findings provide valuable new insights into social learning and cooperative behaviors in adolescents with CP, as well as heterogeneity between adolescents with CP/HCU and those with CP/LCU. They also raise important considerations regarding the need for task development to optimize measures of individual differences in the mechanisms underlying social behaviors in adolescents with CP.

In line with our hypothesis, adolescents with CP (both HCU and CP/LCU) demonstrated reduced ability to differentiate trustworthy’ from untrustworthy environments. This hypothesis was based on prior evidence of reduced cooperation in participants with CP,^5^^,^^24^^,^^34^ as well as the proposal by Fonagy and Luyten^10^ that reduced epistemic trust (or knowledge of whether social communicators are trustworthy) is one key driver of the development of aggressive behaviors such as those seen in CP. An inability to determine who is trustworthy could plausibly lead to a tendency to rely more on aggressive than cooperative strategies when interacting with others, and negatively affect the building and maintaining of social relationships in adolescents with CP. However, although we saw similar degrees of difficulty in differentiating (un)trustworthy social environments across the CP/HCU and CP/LCU groups in our task, this behavioral similarity may stem from at least partially different etiological pathways. CP in the context of LCU appears to be under stronger environmental risk,^40^^,^^41^ with possible links to early life trauma and adversity.^6^^,^^42^ This early adversity could, in turn, disrupt associative learning,^43^ which is likely relevant for socialization over development. CP in the context of HCU is associated with stronger genetic risk^40^^,^^41^ and has been associated with reduced receptivity to social affiliative cues—including cues important for social communication such as eye gaze and happy face.^15^^,^^19^^,^^20^, ^44^, ^45^ This could present a barrier to the ability to learn to differentiate trustworthy and untrustworthy communicators. Adolescents with CP/HCU may also have histories of early adversity, which could exacerbate genetic risk. Future research could elucidate this further by relating differentiation of (un)trustworthy environments in adolescent CP with parent ratings of parent–child relationships and children’s maltreatment history.

Although adolescents with CP/HCU and CP/LCU were considerably poorer than TD adolescents at differentiating (un)trustworthy environments in our study, only adolescents with CP/LCU statistically differed from TD adolescents in their ability to coordinate with social partners—another important aspect of cooperating with others. This is contrary to our hypothesis that coordination in general would characterize adolescents with CP. It is important to note that although adolescents with CP/HCU did not differ statistically from the TD group in their behavior on this task, their behavior did not statistically differ from that of the CP/LCU group either (ie, they were not fully typical in their choices). This suggests that reduced cooperation might relate more to trust environments in adolescents with CP/HCU, and be more general to social decision-making in those with CP/LCU. This would fit with studies suggesting that CP/LCU is associated with poorer social problem solving and flexibility, whereas CP/HCU is more associated with reduced social affiliation.^20^^,^^23^^,^^24^ It is important to note that this difference disappeared when other important factors that are part of the clinical presentation of CP and that differed between groups in our sample (hyperactivity, peer problems, emotional problems) were included as covariates. This implies that broader psychiatric vulnerability that is typically seen in individuals with CP may be important in understanding variance in ability to coordinate between social environments in this group and warrants future investigation.

Interestingly, we observed no group differences in any of the factors that might contribute to cooperative behaviors that we investigated in our study (social preferences, prior beliefs). We had hypothesized that adolescents with CP/HCU might be more willing to accept an advantage over others, and that those with CP (perhaps especially CP/LCU) would be less willing to accept a disadvantage compared to others. Our observed lack of group differences in either of these social preferences does not align with prior research using similar tasks in adolescents with CP.^28^^,^^30^^,^^46^ It is noteworthy that we observed substantial heterogeneity in the responses within each group, and future studies are required to investigate the degree to which sample and task characteristics may affect social preferences. It is also worth noting that inequality aversion represents only one motivational factor that could be driving behavior in our task. Recent research has highlighted that a range of motivations drive individual-level decision making in economic games. These include guilt aversion, greed (maximizing one’s own gain at the expense of others), altruism (avoidance of inequity and guilt) and moral opportunism (flexibly adapting strategy based on the situation).^47^^,^^48^ Indeed, traits associated with psychopathy in adults have been linked to lower levels of reciprocity (which indirectly reflects greed).^49^ Future research that investigates a wider range of motivational factors in relation to social learning and cooperative behaviors in adolescents with CP/CU could be of benefit for understanding social difficulties in this population. Regarding prior beliefs, we hypothesized that adolescents with CP might have reduced expectation that others would cooperate with them, in terms of both trust and social coordination. However, all groups were comparable in their prior beliefs regarding the behavior of unknown others in this task. Given our observation that adolescents with CP had more difficulty differentiating trustworthy and untrustworthy social environments, it would be interesting to extend this work by investigating these groups’ prior beliefs in relation to the behavior of others who should (in theory) be trusted communicators, such as teachers, parents/caregivers, and friends. It should be noted that we were unable to investigate expectation updating because of poor model fit (discussed in more detail below).

Because we did not see group differences in social preferences and prior beliefs, it is difficult to provide a mechanistic explanation of group differences in cooperative behaviors in our study, and it thus leaves room for interpretation. It is possible, for example, that CP participants (perhaps especially those with CP/LCU) were unable to pick up on social information when other, more salient information was present, in this case, one choice being associated with a potentially higher reward. CP has been associated with heightened sensitivity to reward at both a behavioral and neural level.^50^ Future work is needed to further delineate whether salience of social cues (relative to other motivating cues) might modulate the ability of adolescents with CP to take these cues into account and what the consequences of this are to triggering instances of antisocial and aggressive behavior. It is also possible that that our tasks were too complex for our CP participants, which led to more difficulty differentiating social environments. We suggest this is unlikely to be the sole explanation of our findings, as TD participants, who were matched in IQ at a group level with our participants with CP, performed better in both the Trust and the Coordination games than participants with CP. However, it is worth noting that our exploratory analyses indicated that behavior across groups was generally erratic for all of our economic tasks, to the extent that we were unable to fit computational models. To be consistent with prior research, we used the same modeling approach as Westhoff *et al.*,^25^ who used the current task with a large, typically developing, adolescent sample. Although beyond the scope of the current study, systematic exploration of the model space in future studies could advance a mechanistic understanding into belief updating in CP populations, and we suggest that this is best done in conjunction with task development.

It is also important to note that all of our groups had a mean level of IQ that was below average.^51^ Lower cognitive ability is part of the clinical presentation of adolescents with CP, but this may limit the suitability of current tasks and computational models for elucidating the mechanisms underlying atypical behavior in CP. This also raises interesting questions regarding whether current behavioral tasks and computational measures should be further optimized to more sensitively capture the mechanisms underlying social behaviors in adolescents with CP.

Our findings indicate heterogeneity in disrupted cooperative behaviors in adolescents with CP/HCU and CP/LCU. Although adolescents with CP/LCU appeared to struggle to learn to adjust to social environments more generally, those with CP/HCU showed marked differences from TD participants only in relation to trust. These findings provide a strong impetus for future research to explore trust and social learning over development in CP, including relating these to behaviors characteristic of CP, such as aggression. They also raise important questions regarding the best methodology for exploring individual differences in this highly vulnerable group.
