# Supplementary material for: Social Learning and Preferences in Adolescents With Conduct Problems and Varying Levels of Callous-Unemotional Traits
Source: JAACAP Open. 2024 Jan 12;2(2):79–89. doi: 10.1016/j.jaacop.2023.12.008 (PMC11562431; doi:10.1016/j.jaacop.2023.12.008)
Supplement: Supplemental Data [file mmc1.docx]

Supplemental

## *Supplement 1*

Consent procedures

Information sheets were sent to parents of participants, giving them the opportunity to consent to their child participating or to opt their child out of the study. Informed assent was obtained from all child participants prior to participation. Parental consent/opt-out and child assent procedures were in line with General Data Protection Regulations (more information can be found in S1). The current study was approved by the University College London Research Ethics Committee (Project ID number: 0622/001). An ‘opt-out procedure’ was ethically permissible for the current study as the research was non-invasive and in the public interest. Use of an active consent vs opt out procedure was decided on a school-by-school basis at the discretion of the school staff. All child participants received age-appropriate information sheets prior to participation, which were verbally explained, and informed assent was obtained prior to participation.

Group Assignment

## *2.2 Measures*

CP participants were required to meet age-appropriate cut-off scores on the teacher-version of the Child and Adolescent Symptom Inventory (CASI-4R) Conduct Disorder Scale, as outlined in the CASI manual (Gadow & Sprafkin, 2005). The cut-off scores associated with a clinical diagnosis of Conduct Disorder from teacher-report according to the CASI manual are: a score of 3+ (ages 10-12), 4 + (ages 12-14), and 6+ (ages 15-16). A median split approach was then used to separate the children with CP into HCU and LCU groups based on their score on the Inventory of Callous-Unemotional Traits, teacher-version (ICU) (Essau et al., 2006; see ‘Psychometric and questionnaire measures’). For completeness, we also ran main analyses with CU and CP included as continuous covariates (see Table S9). The median ICU score in our CP sample was 38.6. Boys who scored higher than this score were assigned to the CP/HCU group. Boys who scored lower than/equal to this score were assigned to the CP/LCU group. Based on prior published research, 38 represents a clinically meaningful cut-off for HCU for both teacher and parent ratings (Docherty et al., 2017).

To be eligible for the TD group, participants were required to score: (1) below the CP group median on the ICU (i.e. <38.6); (2) within normal range (≤ 2) for the CASI-CD; (3) within normal range (≥ 4) of the prosocial subscale of the Strengths and Difficulties Questionnaire (SDQ); and (4) below the cut-off of 16 for teacher-rated total difficulties (as per SDQ scoring norms; Youth in Mind 2016). Due to missing teacher data, parent data for five TD participants were used for the following measures: CASI-4R, ICU, & SDQ. Full reporting of results of descriptive and demographic analyses can be found in S2.

Psychometric and questionnaire measures

Participants completed the two-subtest version of the Wechsler Abbreviated Scale of Intelligence (WASI; (Wechsler, 1999)), a measure of IQ. We also took a measure of pubertal development (the Pubertal Development Scale (PDS; Carskadon & Acebo, 1993), as our participants were drawn from an age range that encompasses significant developmental change (11-16). This measure showed acceptable internal consistency in our sample (α = 0.73). The Alcohol Use Disorder (AUDIT; Babor et al., 1992)) and Drug Use Disorder (DUDIT; Berman et al., 2005)) tests were also completed to measure substance use. These measures showed good internal consistency in our sample (α = 0.89 and α = 0.83 respectively).

Finally, we took teacher measures of participants’ social and emotional difficulties – or symptoms that commonly co-occur with CP. For these, we used the hyperactivity, emotional problems and peer problems subscales of the Strengths and Difficulties Questionnaire (Goodman, 1997). The measure showed good to very good internal consistency for all sub-scales measured in our sample (emotional problems, 𝛼 = 0.83; peer problems, 𝛼 = 0.92; hyper-activity, 𝛼 = 0.87).

We also saw good to very good reliability for our measures used for group assignment (CASI, α = 0.87; ICU α = 0.93)

Use of Median Split Approach

We employed a median split approach to separate the children with CP to groups with high and lower levels of CU traits (HCU vs. LCU), for the following reasons:

1. Effects of CU traits do not often emerge as interactions and can instead lead to suppressor effects in correlational analyses (Frick, 2012).
2. The median split approach has, in the past, successfully delineated groups of children with CP who have different social-cognitive processing patterns. The pattern of results in these two groups has often been such that, if they had been combined, researchers might have missed deficits in either group (Schwenck et al., 2012; Viding et al., 2012).
3. Suppressor effects can generate difficulties for interpretation, which mean that effects of CU traits may not emerge in interactions, although the CP/HCU and CP/LCU children look very different. The group centric analyses thus make it easier to interpret the translational relevance of findings, which is more challenging when examining suppressor effects in continuous analyses, for example. It is important to note that concerns regarding loss of power from dichotomizing relate to the case of bivariate normality (Cohen, 1983), but using continuous measure of CP and CU can generate problems if modelled together, given the absence of bivariate normality - high CU traits almost invariably denote high levels of CP, but not the other way around (Fontaine et al., 2011)

## *Supplement 2 – Full Descriptive Analyses*

Groups (conduct problems with high callous-unemotional (CP/HCU) traits, CP with low CU traits (CP/LCU), typically developing (TD) were matched in IQ (F(2, 146) = 1.96, p = 0.145, η2 = 0.03), and pubertal stage (χ2 = 11.09, p = 0.182, φc = 0.19) at testing. IQ data were not available for one CP/HCU and one TD participant due to administration error. Analysis of our main models were therefore run with and without these participants, which did not affect key findings (Table S10). Groups differed significantly in Age (F(2, 148) = 3.85, p = 0.023, η2 = 0.05). Post-hoc Bonferroni tests revealed that the CP/LCU group was significantly younger than the CP/HCU group (p = 0.019) but that there were no other group differences (all *ps* > 0.3).

We were unfortunately unable to compare whether groups were similar with respect to ethnicity due to low return rates of parent questionnaires where ethnicity was assessed (data missing per group: CP/HCU = 65%, CP/LCU = 65%, TD = 68%).

As according to group assignment, groups differed significantly on CP. Mann-Whitney U tests revealed that the CP/HCU group had significantly higher CP scores than both CP/LCU (U = 1433.5, p = 0.012, Â = 0.68), and the TD (U = 2714, p = <.0001, Â = 1) groups, and the CP/LCU group scored significantly higher than the TD group (U = 2655, p = <.0001, Â = 0.98). Groups also differed significantly on CU traits as measured by the ICU (F(2, 148) = 225.5, p <.0001; η2 = 0.75). Post-hoc comparisons using the Bonferroni correction indicated that the CP/HCU group scores were higher than CP/LCU (p<.0001), and TD (p<.0001) on the ICU. Scores for the CP/LCU group were higher than for the TD group (p<.0001).

Chi Square tests revealed that groups did not differ significantly on self-reported alcohol use (χ2 = 7.83, p = 0.098, φc = 0.16) or self-reported drug use (χ2 = 5.43, p = 0.066, φc = 0.19).

Groups differed on SDQ rated hyperactivity (F(2, 146) = 66.54, p = <.0001, η2 = 0.48). The CP/HCU had significantly higher hyperactivity scores than the TD group (p >.0001) and the CP/LCU group (p = 0.24), and the CP/LCU group had significantly higher scores than the TD group (p >.0001). Groups also differed on SDQ rated emotional problems. The CP/HCU scored higher than the TD group (U = 1939, p < .0001, Â = 0.714), as did the CP/LCU group (U = 1997.5, p<.0001, Â = 0.736). The CP/HCU and CP/LCU groups did not significantly differ (U=996.5, p = 0.761). Similarly, both CP groups scored higher than the TD group on SDQ rated peer problems (CP/HCU vs TD, U =2077.5, p <0001, Â = 0.77; CP/LCU vs TD, U= 1922, p < .0001, Â = 0.271), but did not differ from each other (U = 1193, p = 0.56).

## *Supplement 3 – Supplementary Methods*

*Economic Games*

Following the procedure of Westhoff et al., (2020), each game comprised 30 trials, each trial involving a one-shot interaction with a new anonymous player. In 15 of these trials, participants interacted with a randomly selected player from the Cooperative environment, while in the remaining 15 trials, they interacted with a randomly selected player from the Uncooperative environment.

It is important to note that: (1) each participant only interacted with 15 of the 20 possible players from each environment, and (2) Westhoff et al. created probabilistic Cooperative and Uncooperative social environments based on real pre-recorded responses on the same games from a sample of Dutch adolescents in the same age range collected by Westhoff et al. for their original study (see Westhoff et al., 2019). These pre-test responses were matched such that cooperative behaviours were displayed by 73% (11/15) of the players in the Cooperative environments of each game, and by 27% (4/15) of the players in the Uncooperative environments (Fig. 1). Choices from other players that were not consistent with their social environment (e.g. an anonymous player from a Cooperative environment making an Uncooperative choice) were fixed on trials 4, 8, 12, and 14 (to be distributed across trials).

In both games participants were required to choose between two options (A or B) to distribute points between themselves and the other player on each round. After having made their own choice, they were shown the choice of the other player (X or Y). Finally, participants were shown the outcome of the combination of their own choice and the other player’s choice, according to the following payoff matrix (where cells in bold represent payoffs for the self, and cells with apostrophes represent payoffs for the other; choices A and B are the top and bottom rows, respectively, and choices X and Y are the left and right columns):

$$\left[ \begin{matrix} \boldsymbol{a}, a' & \boldsymbol{b},b' \\ \boldsymbol{c,} c' & \boldsymbol{d},d' \end{matrix} \right]$$

As each game progressed participants could learn the tendency of their opposing players from each environment to choose X, and adjust their own behaviour accordingly.

In the Trust Game (Fig 1b of the main text), Cooperative and Uncooperative environments were comprised of ‘Trustworthy’ and ‘Untrustworthy’ other players. This game was characterised by the following payoff matrix:

$$\left[ \begin{matrix} \boldsymbol{3},3 & \boldsymbol{1},5 \\ \boldsymbol{2,} 2 & \boldsymbol{2},2 \end{matrix} \right]$$

Participants were able to maximise their earnings by choosing to trust the other player when matched with a person from the Trustworthy environment (option A; top row), and by choosing *not* to trust the other player when matched with a player from the Untrustworthy environment (option B; bottom row).

In the Coordination Game, Cooperative and Uncooperative environments were comprised of ‘Friendly’ and ‘Unfriendly’ other players. This game was characterised by the following payoff matrix:

$$\left[ \begin{matrix} \boldsymbol{2}, 3 & \boldsymbol{0},0 \\ \boldsymbol{0,} 0 & \boldsymbol{3},2 \end{matrix} \right]$$

Participants were able to maximise their earnings by coordinating on the options chosen by other players. In the unfriendly environment, coordinating would typically mean choosing option A, so that participants would accept a disadvantage relative to the other player, but receiving points nonetheless. In the friendly environment, coordinating would typically mean choosing B, so that participants would accept an advantage over that other player.

Games were presented in a fixed order (see ‘Procedure’). The order of trials within each game was presented randomly, but fixed across all participants.

*Non-social Learning Task*


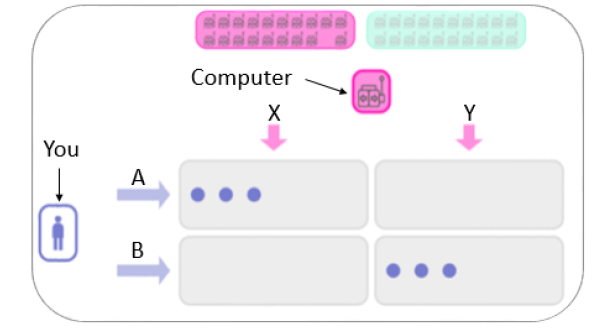


*Fig to accompany S3. Illustration of Non-Social Learning task.*

To determine whether participants were able to adjust behaviour to different environments in a non-social context, we included a learning task where participants were required to two environments with computer opponents. This game was characterised by the following payoff matrix (see accompanying figure):

$$\left[ \begin{matrix} 3 & 0 \\ 0 & 3 \end{matrix} \right]$$

Like in the economic games, participants could maximise their payoffs by coordinating their choices with those of their computer opponent – i.e. choosing option A when playing against a computer from the environment that chooses that most often chooses X (11/15 trials), and B when playing against a computer from the environment that most often chooses Y (11/15 trials).

Learning was incentivised across all tasks by a performance-based gift voucher of up to £7 according to the points participants won.

*Measuring Social Preferences (Dictator Game and Ultimatum Game)*


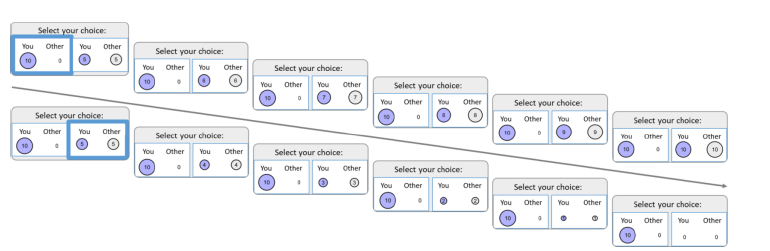


*Figure reproduced with permission from Westhoff et al., (2020) illustrating trial sequence in the Dictator Game. The top sequence = sequence when first choice is for unequal distribution. Bottom panel = choice sequence when first choice is for unequal distribution.*

Social preferences (i.e. advantageous and disadvantageous inequality aversion) were measured in modified versions of two separate tasks: a Dictator Game (DG), and an Ultimatum Game (UG). Both games were adapted to be short and child friendly (based on Blanco et al., 2011; Giamattei et al., 2020).

The DG presented participants with six pairs of binary choices to allocate 10 points between themselves and another anonymous person. One of the options always involved an unequal distribution (10/0) where the participant receives all 10 points and the partner receives none. The other option was an equal distribution of points for both themselves and the partner, with the starting point being (5,5) and decreasing by one point with each subsequent trial [(4,4), (3,3), (2,2), (1,1), (0,0)] or increasing to (10,10) with each subsequent trial [(6, 6), (7, 7), (8, 8), (9, 9), (10, 10)], dependent on the participants’ first choices as follows: if, in the first trial, the participant choose an equal point distribution (5, 5) as opposed to unequal (10,0), the number of points in the equal distribution *decreased* by one point on each subsequent trial (i.e. (5,5), (4,4)…) until (0,0) was reached. If a participant chose the unequal (10,0) distribution in the first trial, the number of points in the equal distribution subsequently increased (i.e. (5,5), (6,6)…) until (10,10) was reached.

We determined participants' advantageous inequality aversion in the DG by identifying the point at which they switched from an equal to unequal distribution preference (i.e. their indifference point, or the point at which they were indifferent between either distribution) (for more detail see S5).


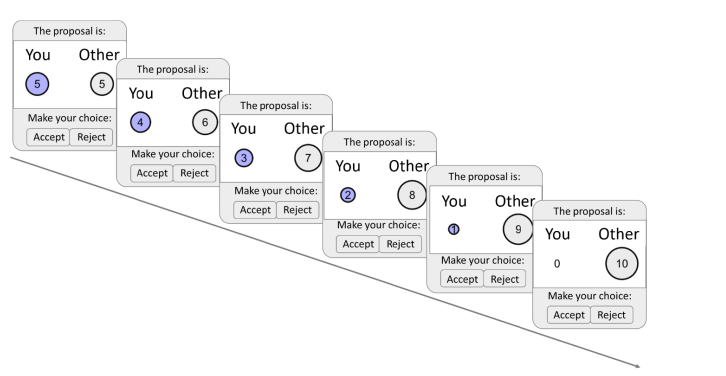


*Figure reproduced with permission from Westhoff et al., (2020) illustrating Ultimatum Game trials (responder stage)*

The UG presented is a sequential two-stage game where participants interacted with another anonymous participant on how to divide 10 points. In the first stage participants acted as the proposer to familiar themselves with the game. In the second round, they were the responder. In both stages, rejecting a proposal resulted in both players earning nothing, while accepting the proposal gave both parties the proposed outcome. In each round of the game, participants were paired with a new player.

Only the data from the ‘responder’ stage of the game were used for subsequent analyses. In this stage, the first proposal was an equal split, while subsequent proposals were increasingly advantageous to the other player - i.e., (5, 5), (4, 6), (3, 7), (2, 8), (1, 9), (0, 10). The point at which participants stopped accepting and started rejecting or vice versa) offers was deemed their ‘minimal acceptable offer’, and this switching point was used to infer participants’ indifference point between the equal and unequal outcome (for more detail see S5).

## *Procedure*

Testing sessions were conducted either in a quiet room on their school premises or at home (5 TD participants). The experiment was programmed in LIONESS Lab (Giamattei et al., 2020), and presented on a Dell Latitude 7480 laptop. Games were presented in a fixed order: (1) Dictator Game, (2) Ultimatum Game, (3) Non-Social Learning Game, (4) Trust Game, (5) Coordination Game. Experimental code is available in editable form from the LIONESS Lab repository .

## *Supplement 4 – Supplemental Analyses*

### Statistical Analyses of Behavioural Data

To analyse choice behaviour in the Trust Game, Coordination Game, and Non-social learning task, we fitted logistic generalized linear mixed models (GLMMs) to decisions to choose A (coded as 1) or

B (coded as 0) for each game separately. Analyses were conducted in R 3.6.167, using the lme4 package (Bates et al., 2015; R. C. Team, 2020; Rs. Team, 2015). All models included the following fixed factors: (1) participant group and (2) environment (Group of Other; other players in the social economic games, and computer groups in the Non-social learning task) on choice behaviour, with their interaction and a subject level random intercept.

### Calculation of social preferences

To determine participants’ *advantageous* inequality aversion, we estimated their indifference points (IPs) in the Dictator Game with a logistic generalized linear mixed model (GLMM) fitted to choices for the equal option (x,x; coded as 1) over the selfish option (10-0; coded as 0), with the size of the share (x) as the fixed effect, and ‘participant’ as random intercept. This model describes the relation between x and participants’ overall preference for choosing the equal option with a logistic function y=(1 / (1 + exp(-Z)), where Z is a linear function b_0_ + b_1_ * x, with two parameters: an intercept (b_0_) and a slope (b_1_). We extracted the individual-specific intercepts b_0_ (i) for each individual and calculated their indifference point by solving for x at y=0.5: IP(i) = -b_0_ (i) / b_1_. Following Blanco et al (2011) and Westhoff et al (2019), a participant’s *i* advantageous inequality aversion is given by β*_i_* = 1 – IP_i_/10.

We determined participants’ *disadvantageous* inequality aversion in a similar way. We first estimated their indifference points in the Ultimatum Game with a logistic GLMM fitted to choices to accept an offer of size x (coded a 1) over choosing to reject it (coded as 0), with the size of the offer (x) as the fixed effect, and ‘participant’ as random intercept. Using the individual-specific intercepts as parameters, we inferred the point at which participants were indifferent between accepting an offer and rejecting it (with both interaction partners receiving 0). This defined their IP_i_. Following Blanco et al (2011) and Westhoff et al (2019), a participant’s *i* disadvantageous inequality aversion is given by α_i_ = IP_i_ / (2 * [5 - IP_i_ ] ).

### Computational Modelling

In order to gain a mechanistic understanding of how participants learned to adjust in the Trust Game and the Coordination Game, we used different versions of a basic reinforcement learning (RL) model (Sutton & Barto, 2018). Please note that results for these models are not reported due to poor model performance (see below).

All models follow the basic logic of RL, where agents learn about others behaviour by updating their expectations via experience. In the Trust and Coordination games, expectations (denoted *p*) concern the behaviour of their interaction partners (X or Y; Figure 1 of main paper), and in the non-social task these expectations concerned the behaviour of groups of computers (see S3). In every trial, the magnitude to which p is updated is proportional to the prediction error (PE; difference between actual and expected choice) and the learning rate λ. Formally, this can be expressed as p_t+1_ = p_t_ + λ · PE, where PE = p – choice of other (1 if X, 0 otherwise). We fit then a set of reinforcement learning models to the data to investigate how λ differs between groups. This λ parameter is bounded between 0 (participants do not update expectations at all) and 1 (an individual’s expectation is updated to fully match the decision of the most recent player).

In our models, the value of p determines the relative weights of A (w_A_) and B (w_B_). Each of the games is characterised by a payoff matrix (see S3). In each trial (t), the expected monetary payoffs of choosing A or B (respectively) are given by w_A,t_ = p_t_ · a + (1 − p_t_) · b and w_B,t_ = p_t_ · c + (1 − p_t_) · d. The initial value of p0 was set to each group’s mean prior measured in our experiment (see ‘Prior Expectations’ in Methods section of main paper). Note that because prior expectations were relatively close to 5 (range 0–10; Fig. 3b) this was close to the default expectation p of 0.5, marking indifference between the environments at the first choice. Hence, we did not apply formal tests of improved model fit for prior expectations.

The probability that a participant chooses A was modelled by a softmax function: Pr(A) = [1 + e^–θ^w_A_ − w_B_)]–1. As participants had to choose between two options (A or B), the probability of choosing B was 1 − Pr(A). In the softmax formula, θ reflects ‘decision sensitivity’ and characterises participants’ choice stochasticity. Low values of θ indicate high levels of stochasticity (Pr(A) and Pr(B) tend to be near 0.5), and high values of θ indicate low levels of stochasticity. In our model fits, θ is a free parameter allowed to vary between 0 and 5.

We extended this baseline model with three factors. First, we include cohort mean values of prior expectations, which determine the initial expectations of a participant at the start of the economic game (p_0_). For models that did not include priors, we assumed p_0_=0.5, indicating that participants believe that their partner would choose X or Y with equal chance. Second, we include the cohort mean measures of social preferences; that is, we add the measured cohort averages of disadvantageous and advantageous inequality aversion to calculate w_A_ and w_B_.. In particular, for the Trust Game, the weight of option A was penalized with a value proportional to the disadvantageous inequality aversion (i.e., α; note that we drop the subscripts as social preferences were assumed to be parameters with a constant value (Fehr & Schmidt, 1999): w_A_ = p_t_ · a + (1 − p_t_) · [ b − α · (b′ − b) ]. As for option B the payoffs for both partners are always equal, w_B_ is unaffected by social preferences. For the Coordination Game, social preferences can affect the weights of both A and B: w_A_ = p_t_ · [a - α · (a′ − a)], and w_B_ = (1 - p_t_) ·[d - β · (d − d′)], where β denotes advantageous inequality aversion. Third, we allowed learning rates to vary between positive and negative prediction errors, so that the model estimated separate parameters for λ_loss_ (used when PE<0) and λ_gain_ (used when PE>0). For each of the three participant groups, we pooled the data and fitted the model with each possible combination of the factors ‘priors’, ‘social preferences’, yielding a total of eight models per group per game.

However, estimated sensitivities in these models were so low that learning rates could not be interpreted for any of our participant groups. Estimates of θ in the best-fitting models were around 0.3, indicating that choices were made almost randomly. This made the estimates of the learnings rates uninterpretable. Different computational models would be unlikely to solve the issue of the low signal-to-noise ratio in our data, and modelling was not the main focus of the current paper. We therefore did not pursue further robustness checks and resorted to a simpler exploratory analysis based on heuristics of win-stay lose-shift (Table S9; described in ‘Results’).

## *Table S1 – Mixed-effects model for the Economic Games*

*Results of binomial generalized linear mixed models (GLMMs) testing effects of (1) participant group and (2) social environment (Group of Other) on choice behaviour in the Trust Game and the Coordination Game.. GLMMs are described fully in S4 (supplemental analyses).*

*Full R-code for the models (this was applied to data from each game):*

*glmer(choice ~ Group*groupOfOther + (1 | ID))*

*family=binomial, na.action=na.exclude, control=glmerControl(optimizer = "bobyqa", optCtrl = list(maxfun = 100000)))*

|  | | | |  |
| --- | --- | --- | --- | --- |
|  | | | |  |
|  | *Decision* | | |  |
|  |  | | |  |
|  |  | | |  |
|  | Trust Game | | Coordination Game |  |
|  | | | |  |
| CP/HCU | 0.499 (0.289) | | 0.447 (0.260) |  |
|  | p = 0.085 | | p = 0.086 |  |
| CP/LCU | 0.585 (0.289) | | 0.588 (0.258) |  |
|  | p = 0.044^*^ | | p = 0.023^*^ |  |
| Group of Other | 0.383 (0.099) | | 0.488 (0.100) |  |
|  | p = 0.0002^***^ | | p = 0.00000^***^ |  |
| CP/HCU x Group of Other | -0.565 (0.153) | | -0.247 (0.151) |  |
|  | p = 0.0003^***^ | | p = 0.101 |  |
| CP/LCU x Group of Other | -0.478 (0.153) | | -0.322 (0.149) |  |
|  | p = 0.002^***^ | | p = 0.031^*^ |  |
| TD Baseline | -0.567 (0.189) | | -1.105 (0.173) |  |
|  | p = 0.003^***^ | | p = 0.000^***^ |  |
|  | | | |  |
| Observations | 4,530 | | 4,530 |  |
| Log Likelihood | -2,972.957 | | -3,027.873 |  |
| Akaike Inf. Crit. | 5,959.913 | | 6,069.747 |  |
| Bayesian Inf. Crit. | 6,004.843 | | 6,114.676 |  |
|  | | | |  |
| *Note:* | | ^*^p<0.05; ^**^p<0.01; ^***^p<0.001 | | |
|  |  | | |  |

Numbers are Estimates with Standard Errors in brackets. CP/HCU - conduct problems and high levels of callous-unemotional traits, CP/LCU - conduct problems and low levels of callous-unemotional traits. TD - typically developing.

## *Table S2 - Mixed-effects model for the Non-Social Task*

*Results of binomial generalized linear mixed models (GLMMs) testing effects of (1) participant group and (2) social environment (Group of Other) on choice behaviour in our Non-social game. GLMMs are described fully in S4 (supplemental analyses).*

*Full R-code for the models is:*

*glmer(choice ~ Group*groupOfOther + (1 | ID))*

*family=binomial, na.action=na.exclude, control=glmerControl(optimizer = "bobyqa", optCtrl = list(maxfun = 100000)))*

|  | | |  |
| --- | --- | --- | --- |
|  | *Decision* | |  |
|  |  | |  |
|  | Non-Social Game | |  |
|  | | |  |
| CP/HCU | 0.331 (0.232) | |  |
|  | p = 0.153 | |  |
| CP/LCU | 0.067 (0.232) | |  |
|  | p = 0.772 | |  |
| Group of Other | 0.724 (0.097) | |  |
|  | p = 0.000^***^ | |  |
| CP/HCU x Group of Other | -0.283 (0.146) | |  |
|  | p = 0.053 | |  |
| CP/LCU x Group of Other | -0.037 (0.146) | |  |
|  | p = 0.798 | |  |
| TD Baseline | -1.196 (0.154) | |  |
|  | p = 0.000^***^ | |  |
|  | | |  |
| Observations | 4,530 | |  |
| Log Likelihood | -3,072.293 | |  |
| Akaike Inf. Crit. | 6,158.586 | |  |
| Bayesian Inf. Crit. | 6,203.516 | |  |
|  | | |  |
| *Note:* | | ^*^p<0.05; ^**^p<0.01; ^***^p<0.001 | |

Numbers are Estimates with Standard Errors in brackets. CP/HCU - conduct problems and high levels of callous-unemotional traits, CP/LCU - conduct problems and low levels of callous-unemotional traits. TD - typically developing.

## *Table S3 - Linear model fitted to participants’ advantageous inequality aversion (see S4 for how we fit our* *advantageous inequality aversion parameter) ..*

*Full R-code for the model:*

*lm(advantageous inequality aversion parameter ~ Group)*

|  | | |  |
| --- | --- | --- | --- |
|  | Dictator Game | |  |
|  |  | |  |
|  | *Advantageous Inequality Aversion* | |  |
|  | | |  |
| CP/HCU | -0.080 | |  |
|  | (0.072) | |  |
|  | p = 0.268 | |  |
|  |  | |  |
| CP/LCU | -0.033 | |  |
|  | (0.072) | |  |
|  | p = 0.649 | |  |
|  |  | |  |
| Constant | 0.465 | |  |
|  | (0.047) | |  |
|  | p = 0.000*** | |  |
|  |  | |  |
|  | | |  |
| Observations | 148 | |  |
| R^2^ | 0.008 | |  |
| Adjusted R^2^ | -0.005 | |  |
| Residual Std. Error | 0.363 (df = 145) | |  |
| F Statistic | 0.620 (df = 2; 145) | |  |
|  | | |  |
| *Note:* | | ^*^p<0.05; ^**^p<0.01; ^***^p<0.001 | |

Numbers are Estimates with Standard Errors in bracketsCP/HCU - conduct problems and high levels of callous-unemotional traits, CP/LCU - conduct problems and low levels of callous-unemotional traits. TD - typically developing.

## *Table S4 – Linear model, fit to disadvantageous inequality aversion in the Ultimatum Game (see S4 for how we fit our* dis*advantageous inequality aversion parameter).*

*Full R-code for the model:*

*lm(disadvantageous inequality aversion parameter ~ Group)*

|  | |
| --- | --- |
|  | *Ultimatum Game:* |
|  |  |
|  | Disadvantageous inequality aversion |
|  | |
| CP/HCU | 0.056 |
|  | (0.139) |
|  | p = 0.687 |
| CP/LCU | 0.071 |
|  | (0.138) |
|  | p = 0.608 |
|  |  |
| Constant | 1.122 |
|  | (0.091) |
|  | p = 0.000*** |
|  | |
| Observations | 148 |
| R^2^ | 0.002 |
| Adjusted R^2^ | -0.012 |
| Residual Std. Error | 0.698 (df = 145) |
| F Statistic | 0.153 (df = 2; 145) |
|  | |
| *Note:* | ^*^p<0.05; ^**^p<0.01; ^***^p<0.001 |

Numbers are Estimates with Standard Errors in brackets. Note: *p<0.1; **p<0.05; ***p<0.01. CP/HCU - conduct problems and high levels of callous-unemotional traits, CP/LCU - conduct problems and low levels of callous-unemotional traits. TD - typically developing.

## *Table S5 – Logistic model fitted to priors (entering the regression as fractions). We observe no group differences in priors.*

*Full R-code for the models (this was applied to estimations for each game):*

*glm(Estimation~Group, family='binomial')*

|  | | |
| --- | --- | --- |
|  | *Priors* | |
|  |  | |
|  | Trust Game | Coordination Game |
|  | | |
| CP/HCU | -0.012 | -0.242 |
|  | (0.394) | (0.398) |
|  | p = 0.977 | p = 0.544 |
|  |  |  |
| CP/LCU | 0.102 | -0.055 |
|  | (0.394) | (0.401) |
|  | p = 0.797 | p = 0.891 |
|  |  |  |
| TD Baseline | 0.081 | 0.434 |
|  | (0.261) | (0.267) |
|  | p = 0.755 | p = 0.104 |
|  |  |  |
|  | | |
| Observations | 151 | 151 |
| Log Likelihood | -107.957 | -105.963 |
| Akaike Inf. Crit. | 221.913 | 217.926 |
|  | | |
| *Note:* | ^*^p<0.05; ^**^p<0.01; ^***^p<0.001 | |

Numbers are Estimates with Standard Errors in brackets. CP/HCU - conduct problems and high levels of callous-unemotional traits, CP/LCU - conduct problems and low levels of callous-unemotional traits. TD - typically developing.

## *Table S6 - logistic GLMM fitted to decisions to ‘stay’, with ‘participant group’, the outcome of the previous interaction (Win vs Lose) with the current group and their interaction as fixed effects, and ‘participant’ as random intercept.*

*Full R-code for the models (this was applied to data from each game):*

*stay ~ Group * win + (1 | id), family='binomial*

|  | *Decision to ‘stick’ with the same choice* | | |
| --- | --- | --- | --- |
|  | Non-social | Trust | Coordination |
|  | | | |
| Win | 0.454 (0.105) | 0.312 (0.107) | 0.085 (0.105) |
|  | p < 0.001*** | p = 0.004*** | p = 0.420 |
| TD baseline | -0.126 (0.110) | 0.113 (0.148) | 0.162 (0.122) |
|  | p = 0.255 | p = 0.447 | p = 0.183 |
| CP/LCU | 0.067 (0.167) | 0.374 (0.226) | -0.130 (0.182) |
|  | p = 0.691 | p = 0.099 | p = 0.478 |
| CP/HCU | 0.229 (0.166) | 0.438 (0.227) | -0.176 (0.184) |
|  | p = 0.169 | p = 0.054 | p = 0.340 |
| CP/LCU x Win | -0.179 (0.159) | -0.139 (0.166) | 0.040 (0.159) |
|  | p = 0.259 | p = 0.404 | p = 0.800 |
| CP/HCU x Win | -0.339 (0.158) | -0.420 (0.167) | 0.275 (0.161) |
|  | p = 0.032* | p = 0.012 * | p = 0.088 |
|  | | | |
| Observations | 4,228 | 4,228 | 4,228 |
| Log Likelihood | -2,830.28 | -2,672.05 | -2,794.93 |
| Akaike Inf. Crit. | 5,674.56 | 5,358.10 | 5,603.85 |
| Bayesian Inf. Crit. | 5,719.00 | 5,402.55 | 5,648.30 |
|  |  |  |  |

*Note* ^*^p<0.05; ^**^p<0.01; ^***^p<0.001

Numbers are Estimates with Standard Errors in brackets. CP/HCU - conduct problems and high levels of callous-unemotional traits, CP/LCU - conduct problems and low levels of callous-unemotional traits. TD - typically developing.

## *Table S7 – Covariate analyses Logistic GLMM fitted to decisions to choose A in Trust game by group of participant (CP/HCU, CP/LCU, TD) and group of other (cooperative or uncooperative) using ‘participant’ as random intercept with covariates.*

*R code for each covariate mode was as follows (models were run separately for each covariate and each game):*

*glmer(choice ~ Group*groupOfOther + COVARIATE + (1 | ID))*

*family=binomial, na.action=na.exclude, control=glmerControl(optimizer = "bobyqa", optCtrl = list(maxfun = 100000)))*

|  | | | | | | |
| --- | --- | --- | --- | --- | --- | --- |
|  | | *Decisions* | | | | |
|  | |  | | | | |
|  | | Trust Game | | | | |
|  | | Original  Model | Age  Covariate | Hyperactivity Covariate | Emotional Problems Covariate | Peer Problems Covariate |
|  | | | | | | |
| CP/HCU | 0.4986 (0.2889) | | 0.4638 (0.2882) | 0.3508 (0.3326) | 0.4433 (0.2977) | 0.4418 (0.3033) |
|  | p = 0.0844 | | p = 0.1075 | p = 0.2916 | p = 0.1364 | p = 0.1452 |
| CP/LCU | 0.5846 (0.2892) | | 0.6297 (0.2889) | 0.4454 (0.3161) | 0.5226 (0.3010) | 0.5102 (0.2985) |
|  | p = 0.0433^*^ | | p = 0.0293^*^ | p = 0.1589 | p = 0.0826 | p = 0.0874 |
| Group of Other | 0.3830 (0.0993) | | 0.3829 (0.0993) | 0.3247 (0.1001) | 0.3247 (0.1001) | 0.3248 (0.1001) |
|  | p = 0.0002^***^ | | p = 0.0002^***^ | p = 0.0012^**^ | p = 0.0012^**^ | p = 0.0012^**^ |
| Age |  | | 0.1112 (0.0571) |  |  |  |
|  |  | | p = 0.0515^*^ |  |  |  |
| SDQ Rated Hyperactivity |  | |  | 0.0122 (0.0305) |  |  |
|  |  | |  | p = 0.6884 |  |  |
| SDQ Rated Emotional Problems |  | |  |  | -0.0131 (0.0306) |  |
|  |  | |  |  | p = 0.6688 |  |
| SDQ Rated Peer Problems |  | |  |  |  | -0.0115 (0.0382) |
|  |  | |  |  |  | p = 0.7631 |
| CP/HCU x Group of Other | -0.5653 (0.1527) | | -0.5652 (0.1527) | -0.5072 (0.1533) | -0.5072 (0.1532) | -0.5072 (0.1533) |
|  | p = 0.0003^***^ | | p = 0.0003^***^ | p = 0.0010^**^ | p = 0.0010^**^ | p = 0.0010^**^ |
| CP/LCU x Group of Other | -0.4776 (0.1527) | | -0.4774 (0.1527) | -0.4217 (0.1543) | -0.4217 (0.1543) | -0.4217 (0.1543) |
|  | p = 0.0018^**^ | | p = 0.0018^**^ | p = 0.0063^**^ | p = 0.0063^**^ | p = 0.0063^**^ |
| TD Baseline | -0.5666 (0.1890) | | -2.1326 (0.8258) | -0.5143 (0.2055) | -0.4669 (0.1950) | -0.4683 (0.1977) |
|  | p = 0.0028^**^ | | p = 0.0099^**^ | p = 0.0124^*^ | p = 0.0167^*^ | p = 0.0179^*^ |
|  | | | | | | |
| Observations | 4,530 | | 4,530 | 4,530 | 4,530 | 4,530 |
| Log Likelihood | -2,972.9570 | | -2,971.0760 | -2,932.3520 | -2,932.3410 | -2,932.3870 |
| Akaike Inf. Crit. | 5,959.9130 | | 5,958.1530 | 5,880.7030 | 5,880.6810 | 5,880.7730 |
| Bayesian Inf. Crit. | 6,004.8420 | | 6,009.5010 | 5,931.9440 | 5,931.9220 | 5,932.0140 |
|  | | | | | | |
| *Note:* | | ^*^p<0.05; ^**^p<0.01; ^***^p<0.001 | | | | |

Numbers are Estimates with Standard Errors in brackets. Estimates are presented to 4 decimal places for clarity (due to similar results). CP/HCU - conduct problems and high levels of callous-unemotional traits, CP/LCU - conduct problems and low levels of callous-unemotional traits. TD - typically developing.

## *Table S8 – Covariate analyses Logistic GLMM fitted to decisions to choose A in Trust game by group of participant (CP/HCU, CP/LCU, TD) and group of other (cooperative or uncooperative) using ‘participant’ as random intercept with covariates.*

*R code for each covariate mode was as follows ( models were run separately for each covariate and each game):*

*glmer(choice ~ Group*groupOfOther + COVARIATE + (1 | ID))*

*family=binomial, na.action=na.exclude, control=glmerControl(optimizer = "bobyqa", optCtrl = list(maxfun = 100000)))*

|  | | | | | |
| --- | --- | --- | --- | --- | --- |
|  | *Decisions* | | | | |
|  |  | | | | |
|  | Coordination Game | | | | |
|  | Original  Model | Age  Covariate | Hyperactivity Covariate | Emotional Problems Covariate | Peer Problems Covariate |
|  | | | | | |
| CP/HCU | 0.4474 (0.2604) | 0.4107 (0.2589) | 0.2281 (0.2851) | 0.2938 (0.2646) | 0.4383 (0.2680) |
|  | p = 0.0857 | p = 0.1127 | p = 0.4237 | p = 0.2669 | p = 0.1020 |
| CP/LCU | 0.5876 (0.2582) | 0.6399 (0.2572) | 0.3631 (0.2741) | 0.3886 (0.2648) | 0.5135 (0.2638) |
|  | p = 0.0229^*^ | p = 0.0129^**^ | p = 0.1853 | p = 0.1423 | p = 0.0517 |
| Group of Other | 0.4885 (0.0998) | 0.4890 (0.0999) | 0.4268 (0.1005) | 0.4267 (0.1005) | 0.4268 (0.1006) |
|  | p = 0.000001^***^ | p = 0.000001^***^ | p = 0.00003^***^ | p = 0.00003^***^ | p = 0.00003^***^ |
| Age |  | 0.1201 (0.0402) |  |  |  |
|  |  | p = 0.0028^**^ |  |  |  |
| SDQ Rated Hyperactivity |  |  | 0.0254 (0.0216) |  |  |
|  |  |  | p = 0.2396 |  |  |
| SDQ Rated Emotional Problems |  |  |  | 0.0331 (0.0216) |  |
|  |  |  |  | p = 0.1255 |  |
| SDQ Rated Peer Problems |  |  |  |  | -0.0344 (0.0272) |
|  |  |  |  |  | p = 0.2058 |
| CP/HCU x Group of Other | -0.2472 (0.1506) | -0.2478 (0.1506) | -0.1854 (0.1510) | -0.1853 (0.1510) | -0.1850 (0.1512) |
|  | p = 0.1007 | p = 0.0998^*^ | p = 0.2198 | p = 0.2199 | p = 0.2210 |
| CP/LCU x Group of Other | -0.3223 (0.1492) | -0.3229 (0.1492) | -0.2378 (0.1507) | -0.2376 (0.1507) | -0.2379 (0.1507) |
|  | p = 0.0308^**^ | p = 0.0305^**^ | p = 0.1146 | p = 0.1149 | p = 0.1145 |
| TD Baseline | -1.1046 (0.1733) | -2.7983 (0.5938) | -1.0835 (0.1826) | -1.0623 (0.1764) | -0.9734 (0.1782) |
|  | p < .001^***^ | p < .001^***^ | p < .001^***^ | p < .001^***^ | p < .001^***^ |
|  | | | | | |
| Observations | 4,530 | 4,530 | 4,530 | 4,530 | 4,530 |
| Log Likelihood | -3,027.8730 | -3,023.5100 | -2,986.8680 | -2,986.3950 | -2,986.7570 |
| Akaike Inf. Crit. | 6,069.7470 | 6,063.0190 | 5,989.7350 | 5,988.7910 | 5,989.5140 |
| Bayesian Inf. Crit. | 6,114.6760 | 6,114.3670 | 6,040.9760 | 6,040.0320 | 6,040.7550 |
|  | | | | | |
| *Note:* | ^*^p<0.05; ^**^p<0.01; ^***^p<0.001 | | | | |
|  |  | | | | |

Table S2.2 - Numbers are Estimates with Standard Errors in brackets. Estimates are presented to 4 decimal places for clarity (due to similar results). CP/HCU - conduct problems and high levels of callous-unemotional traits, CP/LCU - conduct problems and low levels of callous-unemotional traits. TD - typically developing.

## *Table S9 – Model with continuous conduct problems and callous unemotional traits as continuous variables.*

*Numbers are estimates (with SEs in brackets) of a logistic GLMM fitted to decisions to choose A in the Trust and Coordination games respectively by: fixed factors of (1) participant group, (2) group of other, (3) Conduct problems (CP) (4) Callous-unemotional (CU) with the interactions between group of other and CP and group of other and ICU, and a subject level random intercept . All tables produced using ‘Stargazer’ in R* (Hlavac, 2022)*.*

*Full R-code for the models (this was applied to data from each game):*

*glmer(choice1 ~ tCASI*groupOfOther + tICU*groupOfOther + (1 | participantID), family=binomial, data = subset(dat, dat$treatment==2), na.action=na.exclude, control=glmerControl(optimizer = "bobyqa", optCtrl = list(maxfun = 100000)))*

|  | | |
| --- | --- | --- |
|  | *Decision* | |
|  |  | |
|  |  | |
|  | Trust Game | Coordination Game |
|  | | |
| Conduct Problems | 0.040 (0.033) | 0.034 (0.030) |
|  | p = 0.235 | p = 0.257 |
| Group of Other | 0.435 (0.174) | 0.738 (0.173) |
|  | p = 0.013^**^ | p = 0.00003^***^ |
| Callous-unemotional Traits | 0.001 (0.013) | 0.012 (0.012) |
|  | p = 0.938 | p = 0.294 |
| Conduct Problems x Group of Other | -0.040 (0.018) | -0.014 (0.017) |
|  | p = 0.026^**^ | p = 0.415 |
| Callous-unemotional Traits x Group of Other | -0.005 (0.007) | -0.011 (0.007) |
|  | p = 0.484 | p = 0.107 |
| Constant | -0.495 (0.330) | -1.362 (0.300) |
|  | p = 0.134 | p = 0.00001^***^ |
|  | | |
| Observations | 4,530 | 4,530 |
| Log Likelihood | -2,950.558 | -3,003.158 |
| Akaike Inf. Crit. | 5,915.117 | 6,020.316 |
| Bayesian Inf. Crit. | 5,959.999 | 6,065.199 |
|  | | |
| *Note:* | ^*^p<0.1; ^**^p<0.05; ^***^p<0.01 | |

Numbers are Estimates with Standard Errors in brackets. Note: *p<0.1; **p<0.05; ***p<0.01. CP/HCU - conduct problems and high levels of callous-unemotional traits, CP/LCU - conduct problems and low levels of callous-unemotional traits. TD - typically developing.

## *Table S10 – Mixed-effects model for the Economic Games – with participants with missing IQ data removed. This does not change any key findings.*

*Results of binomial generalized linear mixed models (GLMMs) testing effects of (1) participant group and (2) social environment (Group of Other) on choice behaviour in the Trust Game and the Coordination Game.. GLMMs are described fully in S4 (supplemental analyses).*

*Full R-code for the models (this was applied to data from each game):*

*glmer(choice ~ Group*groupOfOther + (1 | ID))*

*family=binomial, na.action=na.exclude, control=glmerControl(optimizer = "bobyqa", optCtrl = list(maxfun = 100000)))*

|  | | |
| --- | --- | --- |
|  | *Decision:* | |
|  |  | |
|  |  | |
|  | Trust Game | Coordination Game |
|  | | |
| CP/HCU | 0.514 (0.292) | 0.399 (0.264) |
|  | p = 0.079 | p = 0.131 |
| CP/LCU | 0.591 (0.291) | 0.611 (0.260) |
|  | p = 0.043^*^ | p = 0.019^*^ |
| Group of Other | 0.399 (0.100) | 0.498 (0.101) |
|  | p = 0.0001^***^ | p = 0.00000^***^ |
| CP/HCU x Group of Other | -0.593 (0.154) | -0.212 (0.152) |
|  | p = 0.0002^***^ | p = 0.165 |
| CP/LCU x Group of Other | -0.494 (0.153) | -0.332 (0.150) |
|  | p = 0.002^***^ | p = 0.027^*^ |
| TD Baseline | -0.573 (0.191) | -1.129 (0.175) |
|  | p = 0.003^***^ | p = 0.000^***^ |
|  | | |
| Observations | 4,470 | 4,470 |
| Log Likelihood | -2,931.697 | -2,983.886 |
| Akaike Inf. Crit. | 5,877.393 | 5,981.773 |
| Bayesian Inf. Crit. | 5,922.229 | 6,026.609 |
|  | | |
| *Note:* | ^*^p<0.05; ^**^p<0.01; ^***^p<0.001 | |

Numbers are Estimates with Standard Errors in brackets. CP/HCU - conduct problems and high levels of callous-unemotional traits, CP/LCU - conduct problems and low levels of callous-unemotional traits. TD - typically developing.
